# Items, Selections and Remarks

**Published:** 1869-09

**Authors:** W. W. Miner


### Items, Selections and Remarks.

BY W. W. MINER, A. B.

Recent writers in the medical journals assert that periosteum, as a covering for the ends of bone in amputation, is of great value. Commencing an inch or so below the point where the bone is to be sawed, the healthy periosteum is dissected back up to that point so as to allow the forming of a flap of periosteum, with which the stump of bone is afterwards covered. In this way a smooth, bony termination will be formed for the stump, and the great tenderness which the ends of amputated limbs often have will be avoided. The proposition appears quite rational.—— Subscriptions from the friends of M. Trousseau, late Professor of Clinical Medicine of the Faculty of Medicine, have been sufficient to furnish two busts of the distinguished physician, one of marble, which is in the Hall of the Faculty of Medicine. The other of bronze, which has been placed on the peristyle of the Hotel Dieu. Each subscriber receives a photographic copy of the busts.——A bi-monthly journal devoted to the study of parasites, "*Zeitschrift fur Parasiten Kunde,*" has been commenced at Jena by Prof. Ernst Hallier, histologist and professor of botany. Each number has two or more lithographic plates, and costs in currency $3.30 each.——A prize of 20,000 livre, offered by Dr. Riberi, of Turin, for the best work on Laryngoscopy, has been received by Prof. Von Bruns, of Tubingen.—— Forty thousand persons were vaccinated in New York, in the month of June, by sixty inspectors appointed by the Board of Health.

E. R. Bickersteth, Surgeon of Liverpool Royal Infirmary, has successfully ligated, by Prof Lister's methods, the carotid artery of a patient for the relief of aneurism. Seven days only, were required for the operation the complete cicatrization of the wound and the discharge of the cured patient. He says, in the *Lancet*, this gives him the opportunity of recording the first cases in which the antiseptic catgut ligature has been successfully used on the human subject. Prof. Lister's reported cases with the catgut ligature have been performed on animals.——A new work in French by Prof. Geraldes, entitled "Clinical Instructions on Surgical Diseases of Children," has just come out, bound in paper, five volumes, which contain, in all, 862 pages. It is compared with Holmes' Surgery as being quite complete and valuable, but it is rather expensive for general circulation.——The pain produced by a blister may be obviated by a hypodermic injection of morphia; and the cicatrization of a blistered surface will be more speedy, if a circular hole is cut in the centre of the blister plaster before it is applied.——Prof. Scanzoni, of Wurzburg, is reported to have received $30,000 in gold for two visits to the Empress of

Russia. Dr. Magni, of Italy, receives one hundred thousand francs for his cataract operation in Lima, Peru.

The common potato, *Solanum Tuberosum*, contains a crystæline base called solanine. This is found in the green leaves of the plant, in the sprouts which receive the sunlight, and in the potatoe tuber itself, when the soil is removed from it so that the sun gives it a green color. Solanine, in doses of more than two grains, produces threatening symptoms. Cattle have been killed by eating of germinated potatoes, such as are used in making spirits. It is, therefore, important, that such potatoes as have grown exposed to the sunlight should be rejected for table use. All the plants of the natural order solanaceæ, among which is the tomato plant, possess this extractive principle.——A Boston drug clerk, who violated his employer's express commands in dispensing any medicine, gave tinct. opii for tinct. rhei, thus causing the death of the patient who, according to directions, took an ounce dose. The officious gentleman is held in bail for one thousand dollars, awaiting the action of the grand jury. Two deaths similarly occasioned have just occurred in Hoboken.

Blackwell's Island Lunatic Hospital now contains twelve hundred patients, twice the number intended to be there accommodated; and the city is building on Ward's Island a new lunatic hospital to accommodate six hundred more, which promises only temporarily to meet the requirements.——The *St. Louis Medical Repertory* recently changed hands, and has since been discontinued because of the loss which attended its publication. The *Medical Archives* is now supplied to the subscribers of the former journal.——Nine, out of seventy pages of the September number of the *Nashville Medical Journal*, are devoted to medical miscellany, the rest to vindication of the editor and personal abuse.

Experiments on the milk of the sow, by Dr. Cameron, a chemist of Dublin, show that it contains eighteen per cent. of nutritious matter, nearly fifty per cent. more than human milk, which contains but eleven. The relative nutritive values of the different kinds is expressed thus in an increasing order; woman's milk, that from the cow, goat, ewe, mare, ass and sow. This accounts for the rapid growth of the young of the pig, and suggests another form of condensed nutriment.——The "golden yellow" hair, fashionable at present, is artificially obtained by applying first a solution of a salt of arsenic, and then brushing on hydro-sulphide of ammonium, which precipitates in the hair, arsenic trisulphide, the well-known golden orpiment. Another method is to get a nitric acid stain by using aqua regia, which is not so pleasant but perhaps quite as safe an application as the former.——The late Philip Maret, of New Haven, bequeathed one-fifth of his property, amounting to one hundred and forty-six thousand dollars, to the Connecticut State Hospital, the income from which is to be used for the support of indigent inmates, especially those whose complaints are incurably established.——A Frenchman in Boston, suffering from a "gum-boil," tried the effects of galvanism in removing the trouble, using a half franc piece and a bit of zinc inserted into his mouth on the parts affected. The result was that the coin slipped into his larynx, where it remained for six weeks, when he applied to Dr. H. K. Oliver, who discovered nothing by means of the laryngoscope; but, as the patient insisted that the coin was there, he placed him on his chest, with his head inclined to the floor, when with blows on his back and continued coughing, the coin was thrown out.

In a Daniell's battery, the porous cell may be dispensed with in this manner: Make the copper plate of such a shape as will allow it to lie upon the bottom of the large cell, place the crystals of cupric sulphate on this plate, fill the jar with the diluted sulphuric acid; the solution of the salt of copper, which is formed, will, by its specific gravity, remain at the lower part of the cell so long as it is undisturbed, while the zinc plates may be suspended in the upper clear portion of the exciting liquid.——Prof. Tiemann of Breslau, uses, in the examination for trichinæ, a lens which magnifies only ten diameters; with a larger diameter, he says the trichinæ may be overlooked.——At the annual meeting of the American Homœopathic Medical Association in New York, this month, Dr. G. D. Beebe, of Illinois, reports that he has removed a portion of intestine four feet ten inches in length, temporarily made use of an artificial anus, then re-established the continuity of the intestinal canal. This account has been noticed also in the daily papers.——A French physician has lately performed Cæsarian section with complete success, in which operation local anæsthia alone was used.——Dr. H. S. Leffingwell, of St. Louis, has used with success the hypodermic injection of caffeine as an antidote to morphia. ——Dr. Braindage, of Factoryville, Pa., reports a case of menstruation occurring regularly in a patient eight years of age.

The University Medical College of New York has just removed to its new building opposite Bellevue Hospital. The building, and its location, are most convenient and desirable ones.——The Kansas State Medical Society has lately been chartered and organized.——Louis Elsberg has been appointed Professor of Diseases of the Larynx, &c., in the Medical Department of the University of New York. He offers two prizes, open for competition to all medical students; one for an illustrated report of the clinic of diseases of the Throat at the college, and another for the best anatomical preparation of the Pharyngo-nasal space.——An Irishman of New York city applied to a lady quack for relief from fever and ague. She administered an infusion of a paper of tobacco in a pint of water. The first dose having produced vomiting, some water and another half-dose were given; the next thing in order was a post-mortem examination, from which it was determined that the man vomited himself to death.

Prof. Von Graefe is in ill health, so as to be obliged to leave his duties in Berlin and to seek in Italy recovery from his invalidism.——Prof. Syme being impaired in health, has resigned the chair of Clinical Surgery in the Edinburgh University; and the students have requested that Prof. Lister, of Glasgow, receive the appointment as his successor.——Prof. Boehm, a noted surgeon of Berlin, died last month from the effects of a dissecting wound.——Dr. C. E. Rider has been appointed lecturer on Ophthalmology in Geneva Medical College.——Rochester University has conferred the degree of L. L. D. on Dr. W. W. Ely of that city.——A prize of twenty thousand dollars from the French Academy for the discovery of the cure for cholera has been waiting for a recipient for fifteen years; in the mean time the income from this amount is expended in encouragement of the study of this malady.

——M. Gonjon has obtained a prize of one hundred dollars from the Academy of Medicine for showing that bone substance may be produced by the transplanting of marrow in the same manner as periosteum. His experiments were on a rabbit, and were quite conclusive.——Wm. Wood & Co. announce that they have in press an American edition of "Holmes' Surgery"; the first of the five volumes will be issued October 1st, and each of the others will follow at intervals of two months.

"At the late meeting of the British Medical Association, Dr. B. W. Richardson exhibited a knife consisting of a working blade, and which divided with such rapidity that superficial incisions could be made with it without pain. The revolutions were about twenty-five per second, bnt the speed might be greatly increased. The knife, in its action, illustrated that an appreciable interval of time is necessary for fixing an impression on the mind and for the development of consciousness. He hoped he should soon be able to give to the surgeon a small pocket instrument with which to open small abscesses, and perform many minor surgical operations painlessly, without having recourse to either general or local anæsthia."—*Lancet.* ——"There seems to be no doubt that the Emperor's condition is more serious than the official journals of Paris represent. The Empress's journey to the East, for which tremendous preparations had been made, has been postponed, and she has hurried back when as far on her way as Corsica. Moreover, Nelaton, the Paris surgeon, who now occupies the position of a last resource to all the wealthy and distinguished people of the continent, has been called in in consultation, and although it is every day announced that his Majesty's indisposition is not serious, and that he will "*soon be about again*," somehow he does not get about. The Bourse, in the meanwhile, is sensitive to the last degree, and ready for a panic of the first order."—*The Nation.*——"M. Nestor Roqueplan was for many years director of the French Opera. If he knows anything, it certainly is Paris and the Parisians, and he has written several works to prove it. His last is "Parisine." Some idea of its spirit may be obtained from the preface to the work. Here it is entire: "*On dit: Strychnine, Quinine, Aniline, Nicotine. Je dis: Parisine.*"—*Nation.*

